# Cost-Effectiveness of Hepatitis E Vaccination Strategies among Patients with Chronic Liver Diseases in China: A Model-Based Evaluation

**DOI:** 10.3390/vaccines12101101

**Published:** 2024-09-26

**Authors:** Fengge Wang, Lu Zhou, Abram L. Wagner, Zixiang Chen, Yihan Lu

**Affiliations:** 1 Shanghai Institute of Infectious Disease and Biosecurity, Fudan University, Shanghai 200032, China; fgwang22@m.fudan.edu.cn (F.W.); zixiangchen23@m.fudan.edu.cn (Z.C.); 2 Department of Epidemiology, Ministry of Education Key Laboratory of Public Health Safety, School of Public Health, Fudan University, Shanghai 200032, China; luzhou22@m.fudan.edu.cn; 3 Department of Epidemiology, School of Public Health, University of Michigan, Ann Arbor, MI 48109, USA; awag@umich.edu; 4 Global Institute for Vaccine Equity, University of Michigan, Ann Arbor, MI 48109, USA

**Keywords:** chronic liver diseases, cost-effectiveness, hepatitis E vaccine

## Abstract

Hepatitis E virus (HEV) is a leading cause of acute viral hepatitis worldwide, primarily transmitted through contaminated water and food. In patients with chronic liver disease (CLD), HEV infection might worsen the prognosis. This study aimed to evaluate the cost-effectiveness of hepatitis E vaccination strategies in CLD patients. A decision tree–Markov cohort model was used to assess the cost-effectiveness of universal-vaccination, vaccination-following-screening, and no-vaccination strategies in 100,000 CLD patients over their lifetimes, simulating cohorts aged ≥16 years, ≥40 years, and ≥60 years, based on the licensed vaccination ages and typical ages of CLD onset, from a societal perspective. Model parameters were retrieved and estimated from previous publications and government data. The outcomes included HEV-related cases, costs, and the incremental cost-effectiveness ratio (ICER). Compared to no-vaccination, universal-vaccination reduced HEV-related cases by 32.8% to 39.6%, while vaccination-following-screening reduced them by 38.1% to 49.3%. Furthermore, universal-vaccination showed ICERs of USD 6898.33, USD 6638.91, and USD 6582.69 per quality-adjusted life year (QALY) for cohorts aged ≥16, ≥40, and ≥60 years, respectively. Moreover, the vaccination-following-screening strategy significantly enhanced cost-effectiveness, with ICERs decreasing to USD 6201.55, USD 5199.46, and USD 4919.87 per QALY for the cohorts. Additionally, one-way sensitivity analysis identified the discount rate and utility for CLD patients as the key factors influencing ICER. Probabilistic sensitivity analysis indicated the vaccination-following-screening strategy was cost-effective with probabilities of 92.50%, 95.70%, and 95.90% for each cohort. Hepatitis E vaccination in CLD patients costs less than GDP per capita for each QALY gained in China. The vaccination-following-screening strategy may be the optimal option, especially in those over 60 years.

## 1. Introduction

Hepatitis E, a liver disease caused by the hepatitis E virus (HEV), is primarily transmitted through the fecal–oral route [1]. Economic growth and improvements in sanitation have led to a decrease in outbreaks associated with genotype 1 of HEV (HEV-1), shifting the epidemiological pattern towards sporadic cases predominantly caused by genotype 4 of HEV (HEV-4) in China [2]. Between 2004 and 2014, the mortality and incidence rates of hepatitis E exceeded those of hepatitis A [3]. HEV infection generally causes a self-limited illness characterized by hepatocyte infection and liver dysfunction. Patients may exhibit no symptoms or only mild symptoms and typically recover quickly. It is estimated that approximately one-third of the global population has been infected at least once in their lifetime, with some individuals maintaining long-term anti-HEV-IgG antibodies in their blood [4]. However, HEV infection can lead to more severe outcomes in certain populations, including patients with chronic liver disease (CLD), the elderly, pregnant women, and immunocompromised patients [5]. Specifically, pregnant women infected with hepatitis E face a 15–25% risk of acute liver failure, with mortality rates potentially reaching up to 30% during the third trimester [6,7]. Immunocompromised patients, such as organ transplant recipients or those living with HIV, are at increased risk for chronic hepatitis E infection, which may progress to cirrhosis, thereby imposing significant health and economic burdens [8,9]. Additionally, the elderly population, especially those with underlying diseases, is at a higher risk for severe complications from HEV infection, including acute and chronic liver failure, leading to an elevated mortality rate [10,11].

In China, the disease burden of CLD is notably high [12]. CLD encompasses a diverse range of conditions, such as chronic hepatitis, cirrhosis, liver cancer, non-alcoholic fatty liver disease (NAFLD), alcoholic liver disease (ALD), and drug-induced liver injury (DILI) [13]. The disease course of CLD is often prolonged, spanning years or even decades, thereby substantially increasing susceptibility to HEV infection. A meta-analysis reported that among hospitalizations with chronic hepatitis B (CHB), the prevalence of HEV superinfection and subsequent mortality were 13.6% and 13.8%, respectively [14]. In CLD patients, HEV infection can significantly exacerbate liver damage, potentially leading to more severe forms of chronic hepatitis and increasing the risk of developing complications such as compensated and decompensated cirrhosis and even liver cancer [11,15,16]. Specifically, the overall rates of liver failure and mortality in CLD patients with HEV superinfection are markedly higher, particularly in those with cirrhosis, indicating significantly increased risks [17]. These findings underscore the critical importance of managing HEV infection in CLD patients, given its potential to accelerate disease progression and elevate mortality risks [8,18].

Administering the hepatitis E vaccine to CLD patients could be a powerful strategy to prevent transmission. The hepatitis E vaccine, HEV 239, has been licensed in China and is administered in three doses following a 0-, 1-, and 6-month schedule for populations aged 16 and older [19,20]. Data from a phase III clinical trial showed that HEV 239 is highly effective in preventing HEV infection, demonstrating a three-dose vaccine efficacy of 100% (95% CI, 72–100%), with only a few mild side effects reported [20]. Additionally, the long-term immunogenicity of the vaccine has been documented to last up to 10 years [21,22]. A well-defined vaccination strategy could aid decision-makers in minimizing economic burdens while effectively controlling the spread of hepatitis E. Current immunization strategies have demonstrated that vaccination-following-screening was the most cost-effective intervention strategy for hepatitis E in the elderly and women of childbearing age [23,24,25]. However, studies specifically focusing on CLD patients infected by HEV remain limited [26].

Considering the recommended vaccination ages and the typical ages of CLD onset, a cost-effectiveness analysis was performed for various initial age cohorts of 16, 40, and 60 years. This study employed a decision tree–Markov model to assess the cost-effectiveness of three hepatitis E vaccination strategies for CLD patients, including universal-vaccination, vaccination-following-screening, and no-vaccination, from a societal perspective in China.

## 2. Materials and Methods

### 2.1. Study Population

This study focused on hepatitis E vaccination in CLD patients. To compare cost-effectiveness, we simulated hepatitis E vaccination in three cohorts of 100,000 CLD patients aged ≥ 16, ≥40, and ≥60 years, based on licensed vaccination ages and the typical ages of CLD onset in China.

### 2.2. Decision Tree–Markov Model

**Figure 1:**
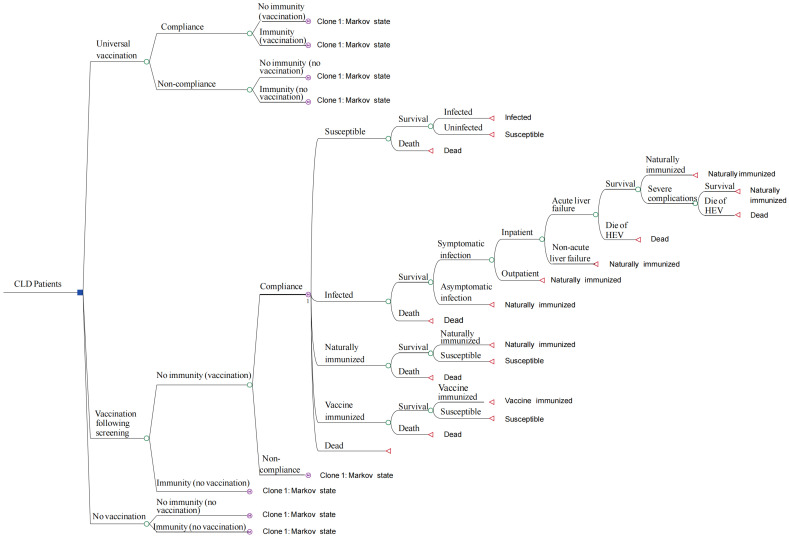
Decision tree–Markov model of HEV vaccination in patients with chronic liver disease. Blue squares represent decision nodes for selecting vaccination strategies. Green circles represent chance nodes, with outcomes determined by probabilities. Red triangles represent terminal nodes, indicating final outcomes. (M) denotes Markov models.

A decision tree–Markov model is effective for visualizing different intervention strategies and tracks changes in health states over time to assess the long-term impact of hepatitis E vaccination [24,27]. In this study, we employed a decision tree–Markov model from a societal perspective, considering all relevant costs and benefits of interventions, including healthcare savings, productivity gains, and overall societal welfare (Figure 1). This study considered patient willingness, offering vaccinations to those who expressed a willingness to receive them. It analyzed three hepatitis E vaccination strategies: (1) The universal-vaccination strategy referred to administering the hepatitis E vaccine to all CLD patients who were willing to receive it. Initially, the model assumed a vaccine-induced immunity probability of vaccine efficacy for recipients, whereas non-recipients were entered into the Markov cycle based on the probability of acquiring natural immunity, with the probability of vaccine immunity being 0. (2) The vaccination-following-screening strategy required comprehensive screening, vaccinating only those who tested negative for anti-HEV antibodies and expressed willingness to be vaccinated. For individuals unwilling to receive the vaccine, the decision tree mirrored that of non-recipients in the universal-vaccination strategy. (3) The no-vaccination strategy, serving as a baseline control, involved no vaccination for CLD patients. The specified vaccination regimen included three doses, administered at 0, 1, and 6 months.

The Markov model was used to simulate the dynamics of HEV infection and its subsequent outcomes, including the states of susceptibility to HEV, infection, natural immunity, vaccine immunity, and death. The model operated on an annual cycle and terminated at the life expectancy of the population, with death defined as the absorbing state (Figure 1). In the analysis, the Markov states were determined as follows: Populations in the susceptible state could transition to the immunized state through vaccination. HEV IgG antibodies, derived from either vaccination or natural infection, diminished over time, leading to a gradual return to susceptibility. Initially, the susceptible population might become infected with HEV, after which they could either recover or progress through the disease process. HEV infection might manifest as either symptomatic or asymptomatic [19,28]. Symptomatic patients might seek outpatient or inpatient medical care, depending on the severity of their medical conditions. Hospitalized patients might progress to either non-acute or acute liver failure, with outcomes varying from survival to death due to HEV infection. Additionally, in CLD patients, hepatitis E infection significantly increased the risk of severe complications and can even lead to death [29]. The decision tree–Markov model was constructed using TreeAge Pro 2022 software (TreeAge Software, Inc., Williamstown, MA, USA).

### 2.3. Model Inputs

The parameters input into the model included transition probabilities among the Markov states, associated costs, and the health-related impacts on quality of life, which were measured with health utility scores. The base values and parameter ranges used in this model were estimated based on published studies and official data provided by government agencies (Table 1).

#### 2.3.1. Probabilities

The willingness to accept vaccines in CLD patients was retrieved based on a survey from Yantai, China, and informed by the parameters utilized in the hepatitis A cost-effectiveness analysis [30,31]. The prevalence of HEV superinfection, the incidence of acute liver failure (ALF), and the case fatality rate of infection were sourced from recent meta-analyses and studies on hepatitis E infection in CLD patients [14,32,33]. Based on the reported number of infection cases and symptomatic cases in a modeling study, we determined the symptomatic rate of hepatitis E infection [34]. The natural immunity rate and the rate of natural immunity decay vary across different age cohorts. To ensure the accuracy and reliability of the parameters, we incorporated data from multiple studies, including long-term epidemiological follow-up studies and similar cost-effectiveness analyses [26,33,35,36,37,38]. The rates of hospitalization and severe complications were derived from studies conducted on hepatitis E in mainland China from 2013 to 2023 [10,17,25,26,33,39]. The case fatality rate associated with severe complications was adopted from established data on hepatitis B [10,40]. The efficacy and duration of protection provided by HEV vaccination were estimated based on results from a phase III clinical trial of the HEV239 vaccine and subsequent long-term follow-up studies [20,21,22]. Furthermore, the all-cause mortality rate for CLD patients was derived from expert opinions expressed in a recent review article on NAFLD [41]. All rates were converted to probabilities using the TreeAge Pro software, employing the formula: *p =* 1 − *e*^−*rt*^.

#### 2.3.2. Costs

The costs incorporated into this model covered both vaccine-related and disease-related expenses. Vaccine-related costs included expenses related to screening, vaccine procurement, indirect costs of vaccination, and administering vaccination services. These vaccine costs were determined based on government procurement prices. The indirect costs were calculated by using the average annual wage of employees in large-scale enterprises in China, converted to a 2-h wage. Disease-related costs included outpatient and inpatient care, as well as costs associated with complications and death. These expenses were derived from recent field surveys among hepatitis E patients in Jiangsu Province, China [26,42]. Additionally, the healthcare costs of managing severe complications were sourced from a survey on the economic burden of diseases related to hepatitis B [33,43]. The per capita Gross Domestic Product (GDP) for the analysis was set at CNY 89,358 (equivalent to USD 12,600.51 based on an exchange rate of USD 1 to CNY 7.1059 as of March 2024) for 2023, based on data from China’s National Bureau of Statistics.

#### 2.3.3. Utility

In this study, quality-adjusted life years (QALYs) were used as the utility metric to evaluate and compare different hepatitis E vaccination strategies. This study encompassed utility values for various states such as susceptibility, asymptomatic HEV infection, outpatient and inpatient cases, as well as severe hepatitis with complications [23,26,33,44,45]. QALYs were calculated by multiplying the number of years lived by the utility value of the quality of life during those years. They account for both the length and quality of life to comprehensively assess the health effects of each strategy. Furthermore, a discount rate of 5% was applied to both cost and utility parameters.

### 2.4. Cost-Effectiveness Analysis

In this study, the decision tree–Markov model facilitated a cost-effectiveness analysis of different hepatitis E vaccination strategies. The outcomes of this study were the incremental cost-effectiveness ratio (ICER), the number of HEV-related cases, and the overall costs. ICER, quantifying the additional cost per QALY, was calculated by comparing the costs and effectiveness of the vaccination strategy versus no vaccination. The willingness-to-pay (WTP) threshold for QALYs was established at GDP per capita, equating to US 12,600.51.

### 2.5. Sensitivity Analysis

This study employed both one-way and probabilistic sensitivity analyses to evaluate the uncertainty of parameter estimates and to ascertain the robustness of the model. One-way sensitivity analysis was conducted to assess the impact of individual model parameters on the ICER by varying each parameter within its specified range of uncertainty while keeping other parameters unchanged, with results displayed in tornado diagrams. Probabilistic sensitivity analysis was performed using Monte Carlo simulations with 1000 random samplings, where all parameters were varied simultaneously according to their probability distributions to evaluate the impact of parameter uncertainty on the ICER. From these simulations, 95% confidence intervals (95%CIs) for the ICER were calculated. The results were visually represented through cost-effectiveness acceptability curves and incremental cost-effectiveness scatterplots.

## 3. Results

### 3.1. Base-Case Results

Compared to the no-vaccination strategy, the universal-vaccination and vaccination-following-screening strategies resulted in reductions of 8273 and 10,637 outpatient cases, 3211 and 4129 inpatient cases, 942 and 1211 cases of ALF, and 177 and 227 deaths, respectively, within the cohort aged 16 years and older. Consequently, these strategies yielded ICERs of USD 6898.33 and USD 6201.55 per QALY, respectively. Both ICER values were lower than GDP per capita, indicating that these vaccination strategies were cost-effective (Table 2). Furthermore, compared with the universal-vaccination strategy, the vaccination-following-screening strategy demonstrated a modest increase in incremental utility and a lower incremental cost. This suggested the vaccination-following-screening was the most optimal strategy. This pattern was consistent in the analysis for the cohort aged 40 years and above, where the vaccination-following-screening strategy led to a reduction of 207 HEV deaths and yielded an improved ICER of USD 5199.46 per QALY (Table 3). These results indicated enhanced cost-effectiveness, particularly in comparison to the younger cohorts. In the cohort aged 60 years and above, the vaccination-following-screening strategy led to a reduction of 173 HEV deaths and an ICER of USD 4919.87 per QALY (Table 4).

Overall, screening before vaccination appeared to improve cost-effectiveness across all age cohorts, with the highest level of cost-effectiveness observed in the cohort aged 60 years and above. The ICER similarly decreased, suggesting that the effects of vaccination became more pronounced in older populations, potentially attributable to their higher baseline risk of HEV-related complications. The number of HEV-related cases that were averted through hepatitis E vaccination gradually decreased as the age at which vaccination began was delayed.

### 3.2. Sensitivity Analysis Results

#### 3.2.1. One-Way Sensitivity Analysis

This study conducted a one-way sensitivity analysis on a range of probability, cost, and utility-related parameters across three cohorts (aged ≥ 16, ≥40, and ≥60 years) (Figures S1–S3). The discount rate and utility for CLD patients emerged as the most influential factors, with the greatest sensitivity to the ICER across all compared strategies. However, even when the remaining parameters fluctuated within their ranges, the ICERs remained below GDP per capita per QALY, indicating that the base-case results were robust.

#### 3.2.2. Probabilistic Sensitivity Analysis

**Figure 2:**
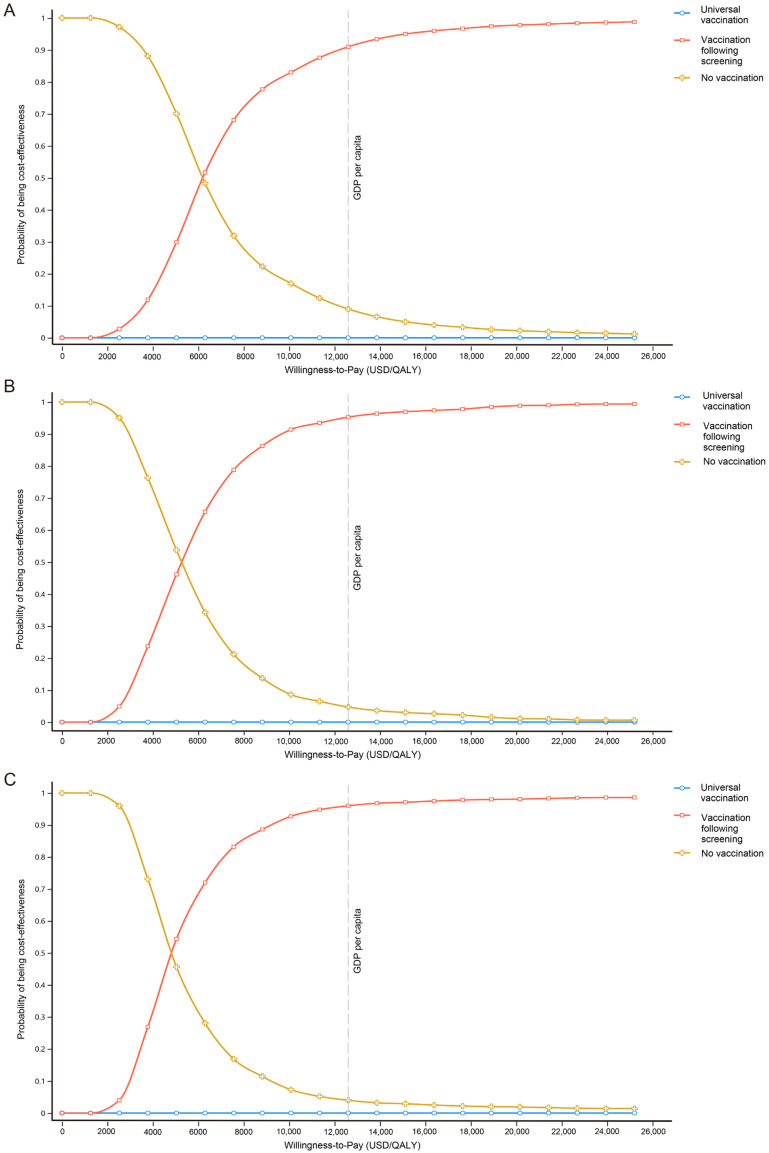
Cost-effectiveness acceptability curves of alternative hepatitis E vaccination strategies. Three cohorts were (**A**) ≥16 years, (**B**) ≥40 years, and (**C**) ≥60 years. At a WTP threshold equal to GDP per capita, the vaccination-following-screening strategy had a probability of being cost-effective of 92.50%, 95.70%, and 95.90% for the three age cohorts, respectively. WTP = China’s GDP per capita (USD) per QALY. Abbreviations: WTP, willingness-to-pay.

The cost-effectiveness acceptability curves for the cohorts exhibited similar trends, indicating consistency with the base-case analysis. Both the universal-vaccination and vaccination-following-screening strategies showed a lower probability of being cost-effective at a WTP value of USD 0. At a WTP threshold equal to GDP per capita, the vaccination-following-screening strategy had a 92.50%, 95.70%, and 95.90% probability of being cost-effective in the three age cohorts, respectively, making it the most favorable option. However, across all three cohorts, the universal-vaccination strategy consistently failed to demonstrate cost-effectiveness (Figure 2).

For each cohort, incremental cost-effectiveness scatterplots displayed a multitude of dots, each representing a range of possible ICERs for each strategy (Figures S4–S6). The scatterplot of incremental cost-effectiveness from 1000 iterations indicated the proportion of cost-effective points for each strategy when comparing any two strategies within each age cohort (Table 5). Compared to the no-vaccination strategy, vaccination strategies were more cost-effective. However, compared to the universal-vaccination strategy, the vaccination-following-screening was more appropriate.

## 4. Discussion

HEV infection significantly threatens the health of CLD patients within the context of the CLD burden in China [15,18]. In our study, findings revealed that hepatitis E vaccination strategies would be cost-effective from a societal perspective when the WTP threshold reached GDP per capita, compared with the no-vaccination strategy. This was consistent with previous studies involving the elderly, women of childbearing age, and CHB patients [23,24,26]. Hepatitis E vaccination typically incurred additional costs but also yielded significant health effects compared to the no-vaccination strategy. Across three cohorts, universal-vaccination reduced outpatient cases, inpatient cases, ALF cases, and deaths by 32.8% to 39.6%. The vaccination-following-screening strategy reduced these outcomes by 38.1% to 49.3% compared to no-vaccination. Moreover, the QALYs gained and costs saved through the vaccination-following-screening strategy surpassed those of the universal-vaccination strategy, consistent across all cohorts aged ≥16, ≥40, and ≥60 years. Awareness of the screening results made individuals more willing to become vaccinated; therefore, the vaccination-following-screening strategy not only improved the health utility of susceptible individuals but also saved vaccine costs for those who are already immune. Consequently, this strategy yielded a more favorable ICER than the universal-vaccination strategy. For certain pathogens, the immune response might differ between natural infection and vaccination; thus, vaccination strategies must consider diverse screening methods. Hepatitis B vaccines are recommended for individuals without a current hepatitis B infection [46]. In contrast, human papillomavirus (HPV) vaccines are widely recommended regardless of past or current infection [47].

Our study evaluated the cost-effectiveness of hepatitis E vaccination in certain populations, supporting its scientific feasibility of implementation. Consequently, the vaccination–following–screening strategy could maximize public health benefits by targeting hepatitis E vaccination in CLD patients. Longitudinal analysis across the three cohorts revealed a trend of decreasing ICERs with age for the vaccination-following-screening strategy. This trend could be attributed to a potential decline in baseline health associated with aging, an increased risk of HEV infection, or a weakened immune system. Previous studies documented a decreasing immune response to influenza and herpes zoster vaccines as exposure increases in middle-aged and elderly populations [48]. This study suggested targeted hepatitis E vaccination strategies for high-risk cohorts may be more cost-effective. However, given the reduction in HEV infections and the incremental gains in QALYs from vaccination, administering the hepatitis E vaccine at an earlier age could protect a larger population. HPV vaccination serves as an example of early-age recommendation to enhance vaccine effectiveness [47]. While HEV infection has similarities with HPV, such as prevalence and limited clinical severity, HEV infection presents a different scenario of moderate duration of protective anti-HEV antibody, which might warrant a trade-off between natural infection and active vaccination. Additionally, resource availability influences the provision of vaccination services across different populations and regions. Therefore, when deciding which age cohorts to prioritize for hepatitis E vaccination in CLD patients, decision-makers must consider factors such as vaccine supply, funding, healthcare facilities, and the feasibility of administering vaccination across different age cohorts.

One-way sensitivity analysis can help decision-makers identify key factors in developing vaccination strategies. In this study, the discount rate and utility for CLD patients were determined to be influential factors across three age cohorts. Additionally, as age increased, the impact of various variables on the ICER diminished. The immune system in middle-aged and elderly individuals may not respond to vaccines as effectively as in younger individuals [49,50]. These findings suggested that age-specific strategies were necessary to optimize the economic viability and health outcomes of HEV vaccination programs. A previous study identified that vaccine coverage, vaccine protection rate, and decay of vaccine protection were crucial in determining the cost-effectiveness of the HEV screening and vaccination strategy for the elderly compared to universal vaccination [23]. Another study highlighted that the cost of vaccination was the main factor influencing its cost-effectiveness among women of childbearing age, particularly when assessing the universal-vaccination versus vaccination-following-screening strategies [24]. Furthermore, probabilistic sensitivity analysis showed that for the cohort aged ≥ 16 years, the vaccination-following-screening strategy had a 92.50% probability of being the most cost-effective option at a WTP threshold of GDP per capita. As age increased, this probability value gradually increased. This facilitated the preparation of appropriate vaccination strategies within budgetary limits and cost-effectiveness criteria for policymakers. Incremental cost-effectiveness scatterplots also showed the distribution of costs and effects within the range of parameter variations when comparing two intervention strategies. Compared to no-vaccination, the vaccination strategies had a higher probability of being cost-effective, as shown by more data points below the WTP threshold line. However, when comparing the universal-vaccination with the vaccination-following-screening strategies, most data points were above the WTP threshold, indicating that vaccination-following-screening was more cost-effective. This cost-saving effect was particularly notable in the cohort aged ≥60 years, likely due to screening effectively identifying individuals who genuinely needed the hepatitis E vaccine, preventing unnecessary vaccinations in the broader population.

Our study had several limitations. First, we assumed that all symptomatic hepatitis E patients received appropriate medical treatment, excluding those unable to access healthcare. This might underestimate the disease burden and overestimate the ICER. Second, this study did not account for the sensitivity and specificity of diagnostic methods, nor did it consider potential vaccine adverse reactions, although previous research has reported very limited adverse reactions [20]. Third, assuming that all individuals willing to receive the hepatitis E vaccine would complete the three-dose regimen might be overly optimistic, potentially overestimating incremental effects and underestimating ICER. Finally, the decision tree–Markov model used in this study did not consider population dynamics or herd immunity effects after vaccination. However, we established three distinct age cohorts, ≥16, ≥40, and ≥60 years based on licensed vaccination ages and typical ages of CLD onset, to effectively compare the cost-effectiveness across different age cohorts. This study also accounted for actual vaccination coverage by considering patient willingness to be vaccinated. Given the complexity of CLD and the potential for severe progression with HEV infection, the decision tree–Markov model included a comprehensive range of disease states, such as cirrhosis and liver cancer. Additionally, we used evidence-based inputs from China to enhance the robustness and reliability of our health economic evaluation.

## 5. Conclusions

In all three age cohorts of CLD patients—those aged ≥ 16, ≥40, and ≥60 years—the vaccination-following-screening strategy proved to be the most cost-effective option in China. It underscores the importance of further developing hepatitis E vaccination strategies to optimize the interventions against HEV infection.

## Figures and Tables

**Table 1:** Base-case values, ranges, and distributions of parameters used in the decision tree–Markov model.

Parameter	Base-Case Value	Range	Reference
Life expectancy	78.2	-	China’s National Bureau of Statistics
**Probability**			
Willing to be vaccinated without screening			
Willingness to be vaccinated after screening	0.800	0.750–0.950	[30]
Infection	0.540	0.492–0.588	[31]
≥16 years	0.136	0.125–0.146	[14,32]
≥40 years	0.190	0.020–0.370	[33]
≥60 years	0.260	0.090–0.420	[33]
Symptomatic infection	0.269	0.209–0.341	[34]
Natural immunity			
≥16 years	0.215	0.127–0.319	[35]
≥40 years	0.408	0.237–0.547	[26,33,36]
≥60 years	0.506	0.329–0.593	[26,33,36]
Natural immunity decay			
≥16 years	0.087	±20%	[37,38]
≥40 years	0.057	±20%	[37,38]
≥60 years	0.081	±20%	[37,38]
Vaccine efficacy	0.866	0.730–0.941	[20,21,22]
Vaccine immunity decay	0.015	0.006–0.031	[21,22]
Hospitalization	0.328	0.273–0.391	[25,26]
Develop into acute liver failure	0.347	0.296–0.401	[14,32]
Develop into severe complications	0.350	0.290–0.410	[10,17,33,39]
Die from severe complications	0.238	0.190–0.286	[10,40]
Die from HEV infection	0.143	0.106–0.185	[14,32]
All-cause mortality of CLD (‰)	15.44	-	[41]
**Cost (USD)**			
Vaccine price (per dose)	109.77	81.62–137.91	Government procurement prices
Screening cost (per time)	4.22	2.11–6.33	Government procurement prices
Vaccination and management costs of vaccination clinics (per dose)	3.94	-	Government procurement prices
Indirect costs of vaccination (per dose)	29.47	-	National Bureau of Statistics
Outpatient cases	101.67	34.53–427.56	[26,42]
Inpatients cases	2867.94	1480.60–4323.39	[42]
Severe complication cases	5842.96	5232.82–6453.10	[33,43]
Death cases	9518.62	6701.12–12336.11	[26]
Vaccination doses	3	-	Default
Discount (%)	5	0–10	Default
GDP per capita	12600.51	-	National Bureau of Statistics
**Utilities (QALY)**			
Health	1	-	Default
Susceptibility	0.79	0.74–0.84	[44]
Asymptomatic HEV cases	0.74	0.70–0.78	Assumption
Outpatient cases	0.70	0.45–0.95	[23,26,45]
Inpatients cases	0.57	0.47–0.63	[33,45]
Severe hepatitis cases and cases with complications	0.38	0.36–0.41	[44]
Death cases	0	-	Default

Abbreviations: CLD, chronic liver disease; QALY, quality-adjusted life year.

**Table 2:** Cost-effectiveness of hepatitis E vaccination strategies in the cohort of patients with chronic liver disease aged 16 years and older.

	Universal-Vaccination	Vaccination-Following-Screening	No-Vaccination
Outpatient cases	16,938	14,574	25,211
Avoided outpatient cases	8273	10,637	-
Inpatient cases	6575	5658	9787
Avoided inpatient cases	3211	4129	-
Cases of ALF	1928	1659	2869
Avoided cases of ALF	942	1211	-
Deaths	361	311	538
Avoided deaths	177	227	-
Total cost (USD)	27,678,535.78	31,260,517.74	13,203,907.74
Incremental cost (USD)	14,474,628.04	18,056,610.00	-
Total QALYs	695,369.94	696,183.29	693,271.66
Incremental QALYs	2098.28	2911.63	-
ICER ^1^	6898.33	6201.55	-
ICER ^2^	4403.99	-	-

^1^ Vaccination strategies (universal-vaccination and vaccination-following-screening) versus no-vaccination. ^2^ Vaccination-following-screening strategy versus universal-vaccination. Abbreviations: ALF, acute liver failure; QALY, quality-adjusted life year; ICER, incremental cost-effectiveness ratio.

**Table 3:** Cost-effectiveness of hepatitis E vaccination strategies in the cohort of patients with chronic liver disease aged 40 years and older.

	Universal-Vaccination	Vaccination-Following-Screening	No-Vaccination
Outpatient cases	14,527	12,680	22,366
Avoided outpatient cases	7840	9687	-
Inpatient cases	5639	4922	8682
Avoided inpatient cases	3043	3760	-
Cases of ALF	1653	1443	2546
Avoided cases of ALF	892	1102	-
Deaths	310	271	477
Avoided deaths	167	207	-
Total cost (USD)	28,103,950.52	28,870,944.05	13,817,212.45
Incremental cost (USD)	14,286,738.07	15,053,731.6	-
Total QALYs	687,329.40	688,072.68	685,177.43
Incremental QALYs	2151.97	2895.25	-
ICER ^1^	6638.91	5199.46	-
ICER ^2^	1031.90	-	

^1^ Vaccination strategies (universal-vaccination and vaccination-following-screening) versus no-vaccination. ^2^ Vaccination-following-screening strategy versus universal-vaccination. Abbreviations: ALF, acute liver failure; QALY, quality-adjusted life year; ICER, incremental cost-effectiveness ratio.

**Table 4:** Cost-effectiveness of hepatitis E vaccination strategies in the cohort of patients with chronic liver disease aged 60 years and older.

	Universal-Vaccination	Vaccination-Following-Screening	No-Vaccination
Outpatient cases	9951	8360	16,451
Avoided outpatient cases	6500	8091	-
Inpatient cases	3863	3245	6386
Avoided inpatient cases	2523	3141	-
Cases of ALF	1133	951	1872
Avoided cases of ALF	740	921	-
Deaths	212	178	351
Avoided deaths	139	173	-
Total cost (USD)	28,015,421.52	27,739,505.33	13,980,739.54
Incremental cost (USD)	14,034,682.00	13,758,765.79	-
Total QALYs	614,569.06	615,233.57	612,437.00
Incremental QALYs	2132.06	2796.57	-
ICER ^1^	6582.69	4919.87	-
ICER ^2^	−415.22	-	-

^1^ Vaccination strategies (universal-vaccination and vaccination-following-screening) versus no-vaccination. ^2^ Vaccination-following-screening strategy versus universal-vaccination. Abbreviations: ALF, acute liver failure; QALY, quality-adjusted life year; ICER, incremental cost-effectiveness ratio.

**Table 5:** ICERs with 95% CI from Monte Carlo simulation results and probability of being cost-effective in the incremental cost-effectiveness scatterplot across different age cohorts.

	Universal-Vaccination vs. No-Vaccination	Vaccination-Following-Screening vs. No-Vaccination	Vaccination-Following-Screening vs. Universal-Vaccination
≥16 years			
ICER (95%CI)	9927.83 (3208.86, 19,955.95)	8816.25 (2900.71, 17,574.03)	6234.49 (2000.14, 12,785.76)
Probability (%)	91.3	93.9	97.4
≥40 years			
ICER (95%CI)	7348.40 (2870.84, 18,780.37)	5647.61 (2218.23, 13,958.04)	1338.87 (−745.48, 4556.72)
Probability (%)	88.8	94.9	99.9
≥60 years			
ICER (95%CI)	7817.78 (3259.08, 19,655.87)	5715.58 (2308.10, 14,581.01)	−215.89 (−3971.33, 2358.74)
Probability (%)	92.0	96.6	99.9

Abbreviations: 95% CI, 95% confidence interval.

## Data Availability

The datasets used and/or analyzed during the current study are available from the corresponding author upon reasonable request.

## Supplementary Materials

The following are available online at https://www.mdpi.com/article/10.3390/vaccines12101101/s1, Figure S1: Tornado diagram for one-way sensitivity analysis of hepatitis E vaccination cost-effectiveness comparing universal-vaccination vs. no-vaccination. Panels represent analyses for (A) cohort ≥16 years, (B) cohort ≥40 years, and (C) cohort ≥60 years. Abbreviations: CLD, chronic liver disease; ICER, Incremental cost-effectiveness ratio. Figure S2: Tornado diagram for one-way sensitivity analysis of hepatitis E vaccination cost-effectiveness comparing vaccination-following-screening vs. no-vaccination. Panels represent analyses for (A) cohort ≥16 years, (B) cohort ≥40 years, and (C) cohort ≥60 years. Abbreviations: CLD, chronic liver disease; ICER, Incremental cost-effectiveness ratio. Figure S3: Tornado diagram for one-way sensitivity analysis of hepatitis E vaccination cost-effectiveness comparing universal-vaccination vs. vaccination-following-screening. Panels represent analyses for (A) cohort ≥16 years, (B) cohort ≥40 years, and (C) cohort ≥60 years. Abbreviations: CLD, chronic liver disease; ICER, Incremental cost-effectiveness ratio. Figure S4: Incremental cost-effectiveness scatterplots of the cost-effectiveness probability sensitivity analysis comparing universal-vaccination vs. no-vaccination for different age cohorts. Panels represent analyses for (A) cohort ≥16 years, (B) cohort ≥40 years, and (C) cohort ≥60 years. WTP = China’s GDP per capita (USD) per QALY. Abbreviations: WTP, Willingness-to-pay; ICER, Incremental cost-effectiveness ratio. Figure S5: Incremental cost-effectiveness scatterplots of the cost-effectiveness probability sensitivity analysis comparing vaccination-following-screening vs. no-vaccination for different age cohorts. Panels represent analyses for (A) cohort ≥16 years, (B) cohort ≥40 years, and (C) cohort ≥60 years. WTP = China’s GDP per capita (USD) per QALY. Abbreviations: WTP, Willingness-to-pay; ICER, Incremental cost-effectiveness ratio. Figure S6: Incremental cost-effectiveness scatterplots of the cost-effectiveness probability sensitivity analysis comparing universal-vaccination vs. vaccination-following-screening for different age cohorts. Panels represent analyses for (A) cohort ≥16 years, (B) cohort ≥40 years, and (C) cohort ≥60 years. WTP = China’s GDP per capita (USD) per QALY. Abbreviations: WTP, Willingness-to-pay; ICER, Incremental cost-effectiveness ratio.
