# Supplementary material for: Cost-Effectiveness of Hepatitis E Vaccination Strategies among Patients with Chronic Liver Diseases in China: A Model-Based Evaluation
Source: Vaccines (Basel). 2024 Sep 26;12(10):1101. doi: 10.3390/vaccines12101101 (PMC11511531; doi:10.3390/vaccines12101101)
Supplement: Supplementary file 1 [file vaccines-12-01101-s001.zip › Supplementary Materials_20240911.pdf]

## Supplementary Materials

### Figure legends

Figure S1. Tornado diagram for one-way sensitivity analysis of hepatitis E vaccination cost-effectiveness comparing universal-vaccination vs. no-vaccination. Panels represent analyses for (A) cohort ≥16 years, (B) cohort ≥40 years, and (C) cohort ≥60 years. Abbreviations: CLD, chronic liver disease; ICER, Incremental cost-effectiveness ratio.

Figure S2. Tornado diagram for one-way sensitivity analysis of hepatitis E vaccination cost-effectiveness comparing vaccination-following-screening vs. no-vaccination. Panels represent analyses for (A) cohort ≥16 years, (B) cohort ≥40 years, and (C) cohort ≥60 years. Abbreviations: CLD, chronic liver disease; ICER, Incremental cost-effectiveness ratio.

Figure S3. Tornado diagram for one-way sensitivity analysis of hepatitis E vaccination cost-effectiveness comparing universal-vaccination vs. vaccination-following-screening. Panels represent analyses for (A) cohort ≥16 years, (B) cohort ≥40 years, and (C) cohort ≥60 years. Abbreviations: CLD, chronic liver disease; ICER, Incremental cost-effectiveness ratio.

Figure S4. Incremental cost-effectiveness scatterplots of the cost-effectiveness probability sensitivity analysis comparing universal-vaccination vs. no-vaccination for different age cohorts. Panels represent analyses for (A) cohort ≥16 years, (B) cohort ≥40 years, and (C) cohort ≥60 years. WTP = China's GDP per capita (USD) per QALY. Abbreviations: WTP, Willingness-to-pay; ICER, Incremental cost-effectiveness ratio.

Figure S5. Incremental cost-effectiveness scatterplots of the cost-effectiveness probability sensitivity analysis comparing vaccination-following-screening vs. no-vaccination for different age cohorts. Panels represent analyses for (A) cohort ≥16

years, (B) cohort ≥40 years, and (C) cohort ≥60 years. WTP = China's GDP per capita (USD) per QALY. Abbreviations: WTP, Willingness-to-pay; ICER, Incremental cost-effectiveness ratio.

Figure S6. Incremental cost-effectiveness scatterplots of the cost-effectiveness probability sensitivity analysis comparing universal-vaccination vs. vaccination-following-screening for different age cohorts. Panels represent analyses for (A) cohort ≥16 years, (B) cohort ≥40 years, and (C) cohort ≥60 years. WTP = China's GDP per capita (USD) per QALY. Abbreviations: WTP, Willingness-to-pay; ICER, Incremental cost-effectiveness ratio.
