# Supplementary figures and images for: Cost-Effectiveness of Hepatitis E Vaccination Strategies among Patients with Chronic Liver Diseases in China: A Model-Based Evaluation
Source: Vaccines (Basel). 2024 Sep 26;12(10):1101. doi: 10.3390/vaccines12101101 (PMC11511531; doi:10.3390/vaccines12101101)

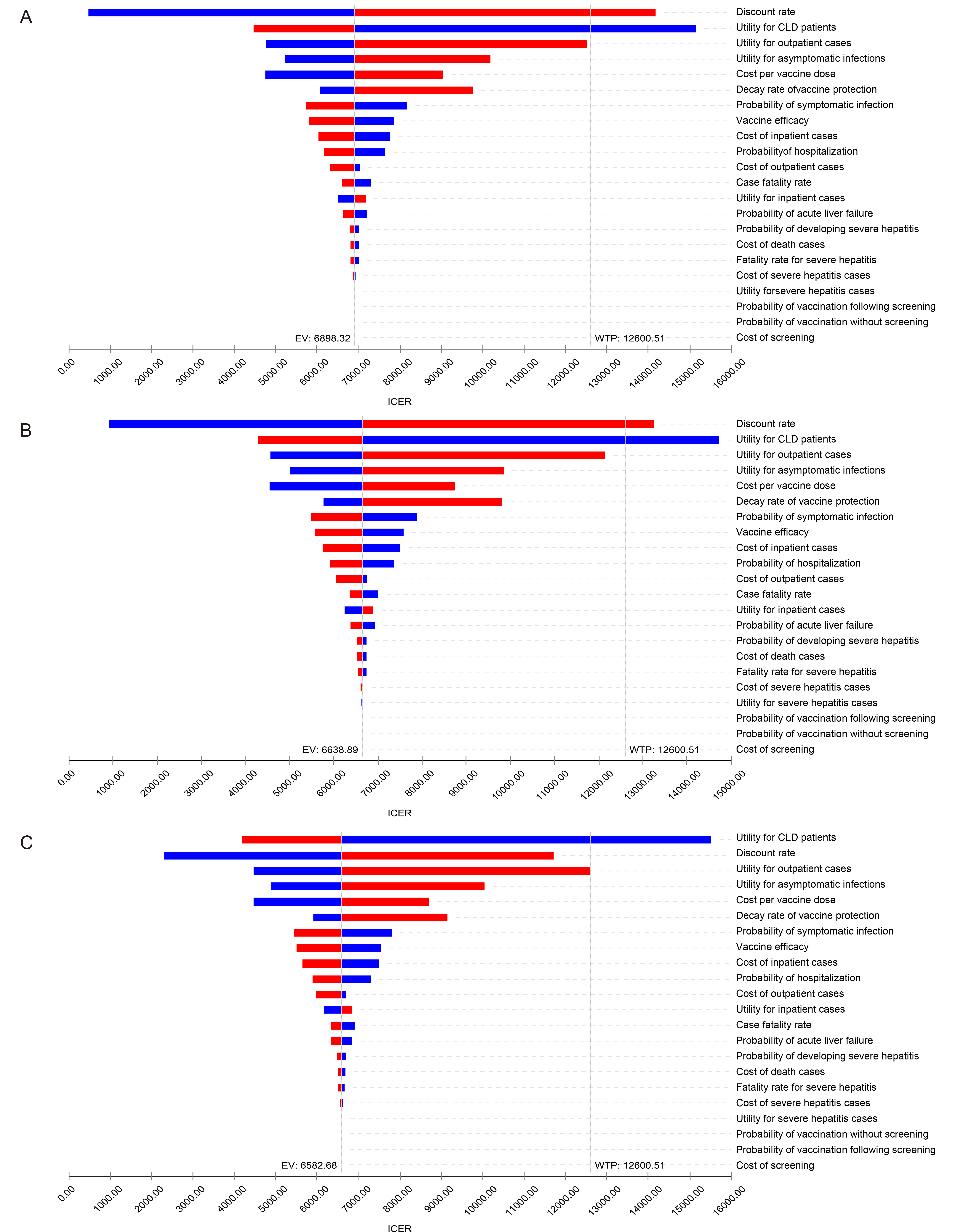

Supplement: Supplementary file 1 [file vaccines-12-01101-s001.zip › FigureS1.tif]

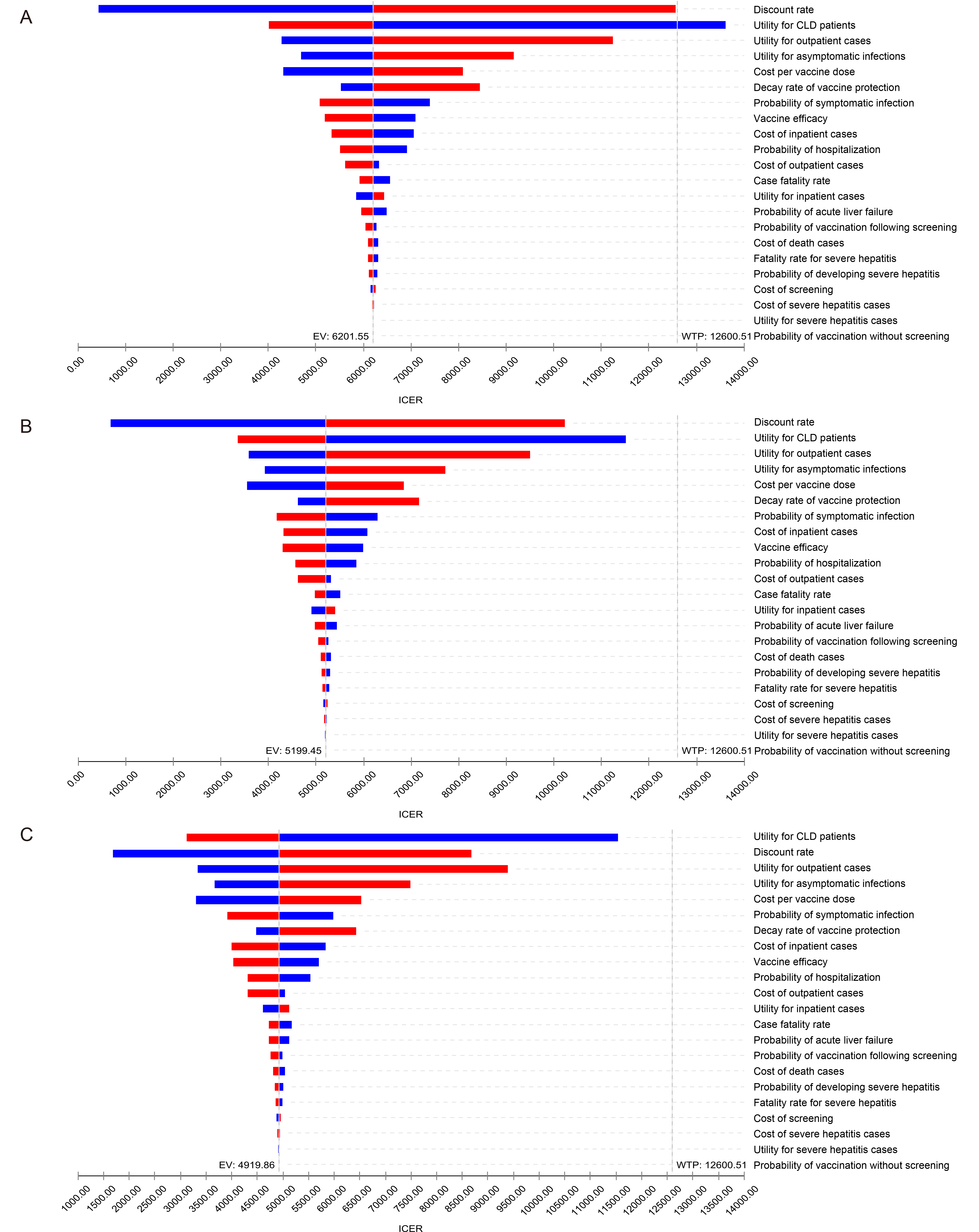

Supplement: Supplementary file 1 [file vaccines-12-01101-s001.zip › FigureS2.tif]

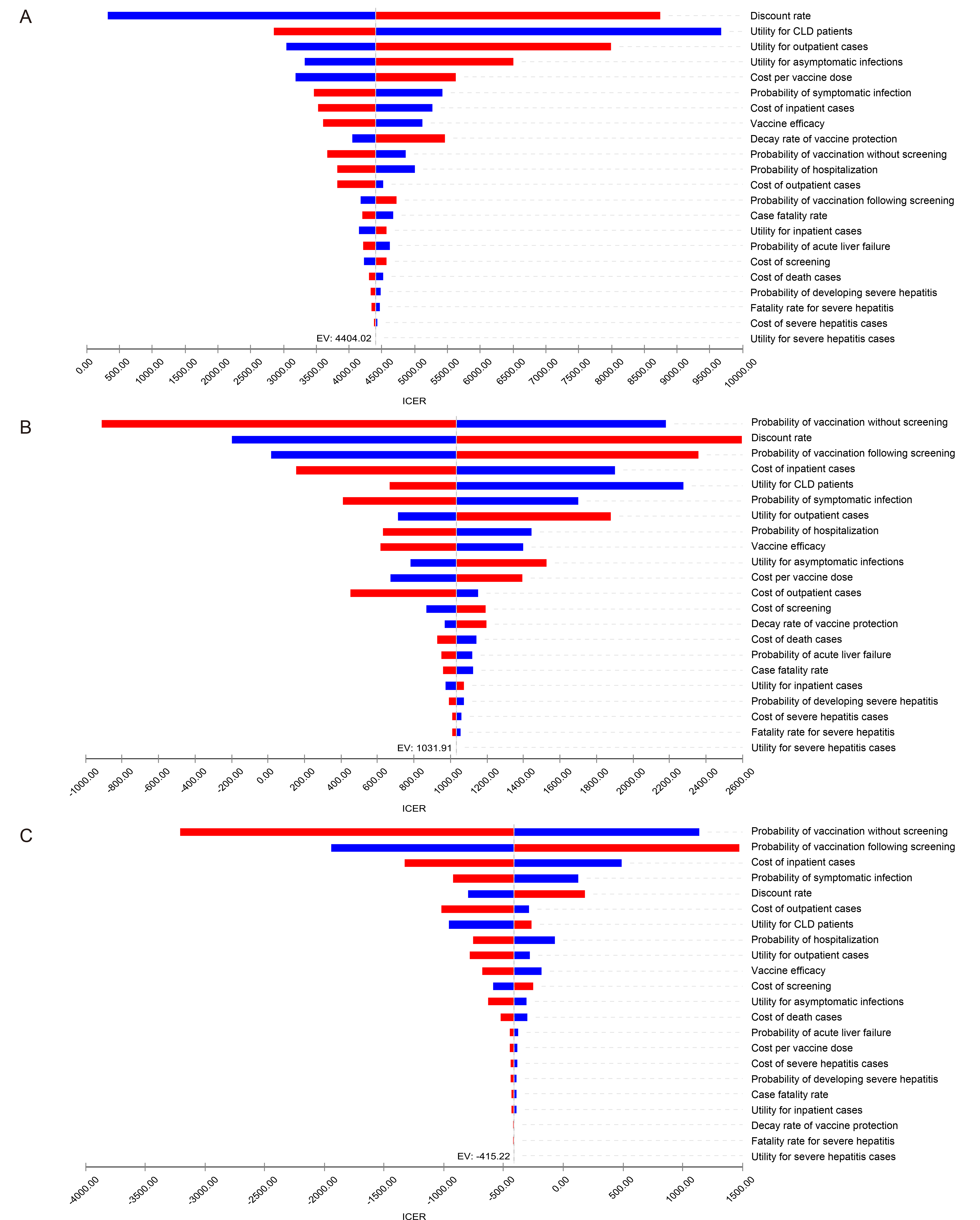

Supplement: Supplementary file 1 [file vaccines-12-01101-s001.zip › FigureS3.tif]

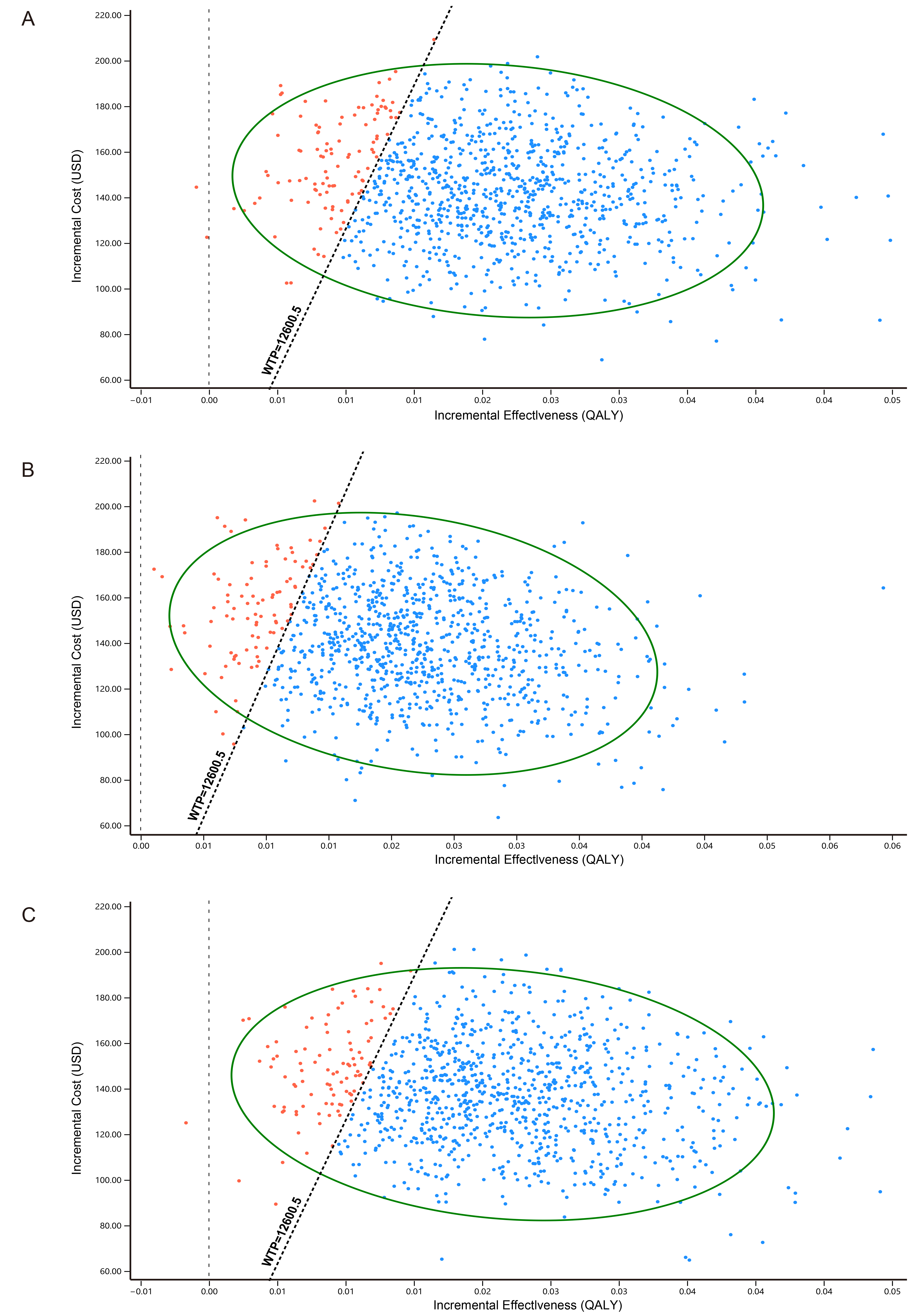

Supplement: Supplementary file 1 [file vaccines-12-01101-s001.zip › FigureS4.tif]

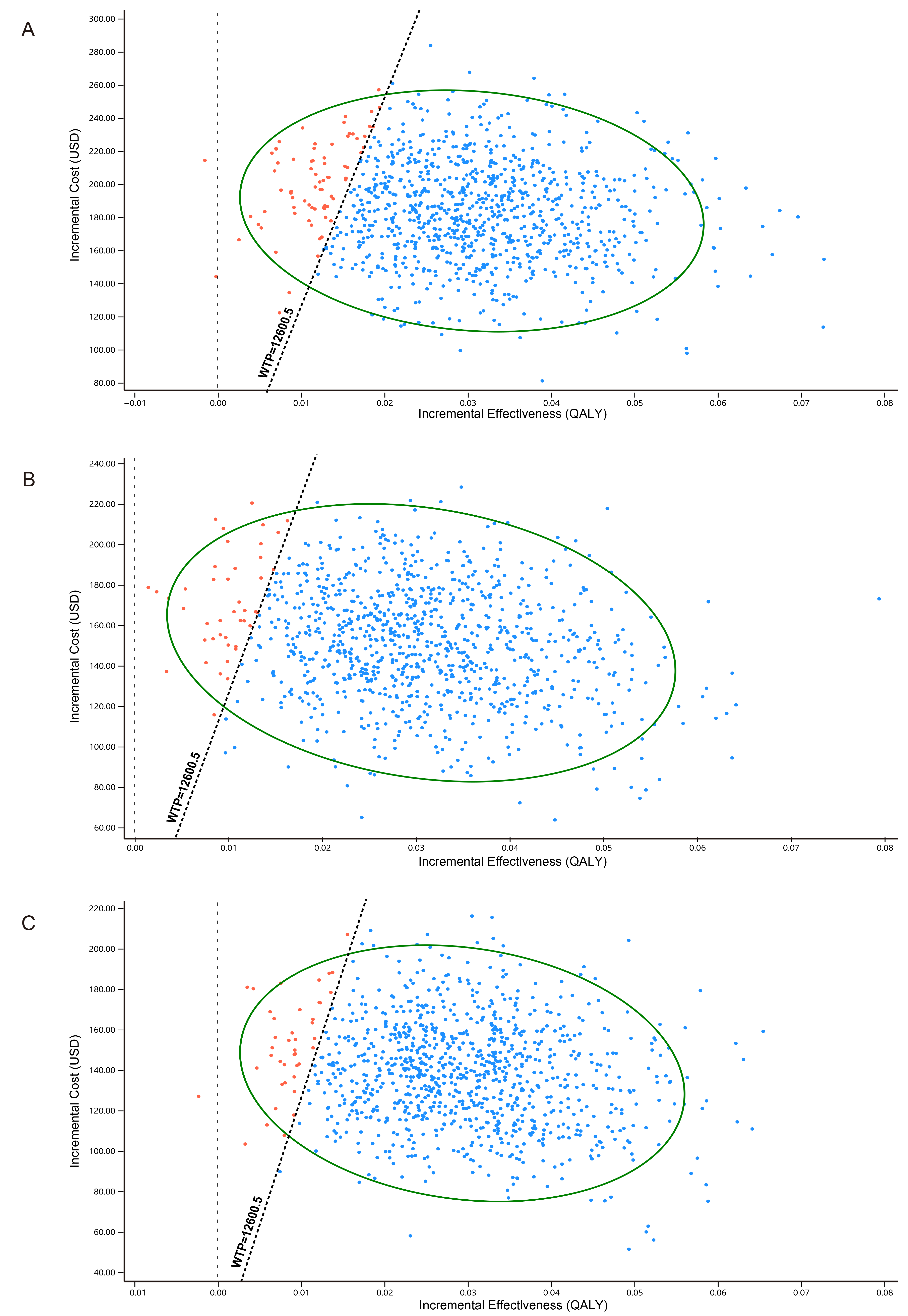

Supplement: Supplementary file 1 [file vaccines-12-01101-s001.zip › FigureS5.tif]

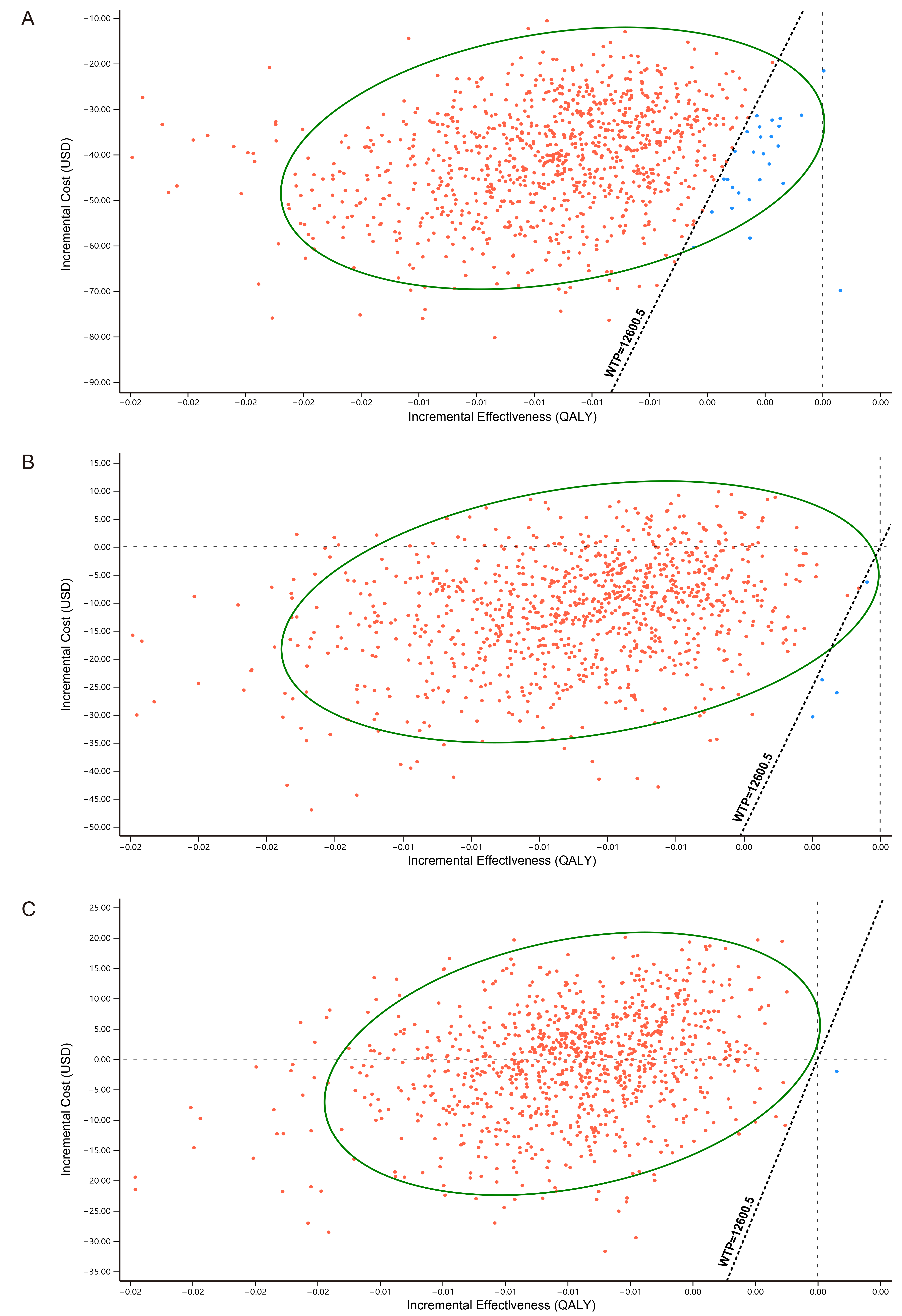

Supplement: Supplementary file 1 [file vaccines-12-01101-s001.zip › FigureS6.tif]
